# Associations between speech understanding and auditory and visual tests of verbal working memory: effects of linguistic complexity, task, age, and hearing loss

**DOI:** 10.3389/fpsyg.2015.01394

**Published:** 2015-09-16

**Authors:** Sherri L. Smith, M. Kathleen Pichora-Fuller

**Affiliations:** ^1^Audiologic Rehabilitation Laboratory, Auditory Vestibular Research Enhancement Award Program, Veterans Affairs Medical Center, Mountain Home, TNUSA; ^2^Department of Audiology and Speech-Language Pathology, East Tennessee State University, Johnson City, TNUSA; ^3^Department of Psychology, University of Toronto, Mississauga, ONCanada; ^4^Toronto Rehabilitation Institute, University Health Network, Toronto, ONCanada; ^5^Rotman Research Institute, Baycrest Hospital, Toronto, ONCanada; ^6^Linneaus Centre HEAD, Linköping University, LinköpingSweden

**Keywords:** hearing loss, speech understanding, aging, reading working memory, listening working memory, speech-in-noise

## Abstract

Listeners with hearing loss commonly report having difficulty understanding speech, particularly in noisy environments. Their difficulties could be due to auditory and cognitive processing problems. Performance on speech-in-noise tests has been correlated with reading working memory span (RWMS), a measure often chosen to avoid the effects of hearing loss. If the goal is to assess the cognitive consequences of listeners’ auditory processing abilities, however, then listening working memory span (LWMS) could be a more informative measure. Some studies have examined the effects of different degrees and types of masking on working memory, but less is known about the demands placed on working memory depending on the linguistic complexity of the target speech or the task used to measure speech understanding in listeners with hearing loss. Compared to RWMS, LWMS measures using different speech targets and maskers may provide a more ecologically valid approach. To examine the contributions of RWMS and LWMS to speech understanding, we administered two working memory measures (a traditional RWMS measure and a new LWMS measure), and a battery of tests varying in the linguistic complexity of the speech materials, the presence of babble masking, and the task. Participants were a group of younger listeners with normal hearing and two groups of older listeners with hearing loss (*n* = 24 per group). There was a significant group difference and a wider range in performance on LWMS than on RWMS. There was a significant correlation between both working memory measures only for the oldest listeners with hearing loss. Notably, there were only few significant correlations among the working memory and speech understanding measures. These findings suggest that working memory measures reflect individual differences that are distinct from those tapped by these measures of speech understanding.

## Introduction

For over a half century, researchers and clinicians have recognized that speech understanding difficulties are common amongst older listeners, particularly when speech is presented in a noisy background or when listeners have age-related hearing loss (e.g., Bocca and Calearo, 1963; Frisina and Frisina, 1997; Gates and Mills, 2005; Mills et al., 2006; Humes and Dubno, 2010). It is well known that both sensory and cognitive processes are independently and interactively involved in speech understanding (e.g., Committee on Hearing, Bioacoustics, and Biomechanics [CHABA], 1988). Research examining the interactions between sensory and cognitive processes has resulted in the emerging field of cognitive hearing science, with much of the recent work in this field focusing on the role that working memory plays in speech understanding in listeners who may have various degrees and types of hearing loss (Arlinger et al., 2009). Working memory is thought to be important for speech understanding because listeners must decode the incoming speech signal while relating the information to stored knowledge and anticipating the speech that is forthcoming (e.g., Daneman and Carpenter, 1980, 1983; Pichora-Fuller et al., 1995; Daneman and Merikle, 1996; Wingfield and Stine-Morrow, 2000; Akeroyd, 2008). When the audibility of the speech signal is reduced due to hearing loss or noise, then more working memory resources may need to be allocated when listeners are trying to comprehend the impoverished incoming speech signal (see also Rabbitt, 1968, 1991; van Rooij and Plomp, 1990; Wingfield, 1996; Lunner, 2003; Humes and Floyd, 2005; Foo et al., 2007; Rudner et al., 2007, 2011; Akeroyd, 2008; Parbery-Clark et al., 2009; Besser et al., 2013). Converging evidence from studies associating working memory measures to speech recognition measures (e.g., measures of how accurately words are repeated by listeners) suggests that inter-individual differences in working memory span explain a small portion of the variance and that listeners with high working memory span have better speech recognition in adverse listening conditions relative to those with low working memory capacity (see Akeroyd, 2008; Besser et al., 2013; and Humes et al., 2013 for reviews). Some studies, however, have been more successful than others in associating working memory and speech-recognition measures, perhaps in part due to the variations in the working memory and speech measures used.

Some researchers have suggested that when examining the associations between working memory and speech understanding, working memory measures should be presented in the visual domain to avoid potential sensory encoding issues associated with the auditory presentation of materials, particularly for listeners with hearing loss (e.g., Souza, 2012). Others have suggested, however, that because working memory is both domain- and modality-specific, it may be more appropriate to measure working memory using test materials presented in conditions that approximate the functional situation of interest (e.g., Pichora-Fuller et al., 1995; Baldwin and Ash, 2011; Besser et al., 2013; for a review of the issue of modality-specificity in testing auditory processing see Cacace and McFarland, 2013). In other words, to understand better the interplay of working memory and speech understanding in everyday listening conditions, it may be better to test working memory using auditory verbal stimuli. Both auditory and visual working memory tests have been used in recent studies, but the reading span measure has been the most commonly used in studies examining the association between working memory with speech recognition (see Akeroyd, 2008; Besser et al., 2013). Of the studies that included auditory working memory tests, few directly compared reading and listening working memory measures in relation to speech recognition in the same sample. It is important to compare the association between auditory and visual tests of verbal working memory and various measures of speech understanding in different listener groups before deciding on how specific test(s) could be used by rehabilitative audiologists. Of course, testing reading working memory rather than listening working memory to assess inter-individual differences in speech understanding would be a reasonable choice if reading and listening working memory tests yielded similar results, but assumptions about the modality-independence of working memory based on research in normal young listeners need to be confirmed in older adults and in listeners who have various degrees and types of hearing loss.

Mixed findings have been reported in a series of three recent Dutch studies examining the associations between measures of reading (Dutch version of the Daneman and Carpenter, 1980 test) and listening span (an auditory version of their reading span version presented in quiet) and a sentence-in-noise repetition task (Versfeld et al., 2000) in younger or middle-aged adults. In the first study (Koelewijn et al., 2012), middle-age listeners (*n* = 32; mean age = 51.3 years) with normal hearing were tested and a significant correlation between the reading and listening span measures (Pearson *r* = 0.67) was found; there also were significant correlations between reading span and sentence recognition thresholds in fluctuating and single-talker maskers (Pearson *r* = -0.36 to -0.50), but no significant correlations between listening span and the sentence-recognition thresholds. In another study using the same Dutch measures in younger adults (*n* = 24) with normal hearing (Zekveld et al., 2013), no significant correlations between the two span measures were found and neither span measure correlated significantly with the scores on the sentence-in-noise repetition task. However, in a third study (Besser et al., 2013) using the Dutch measures in younger listeners with normal hearing (*n* = 42) in two sessions (test–retest purposes), there was a significant correlation between the span measures administered in the two modalities (Pearson *r* = 0.49 in session 1 and *r* = 0.60 in session 2), but neither span measure correlated with speech-in-noise performance in either session. Taken together, these studies suggest that measures of reading and listening span in quiet are usually significantly correlated, but correlations between span measures and speech-in-noise thresholds for speech recognition are elusive for reading span and absent for listening span in quiet when younger or middle-aged adults with normal audiometric thresholds are tested. It is possible that little working memory resources are required by these listeners in these test conditions.

In contrast, studies comparing younger adults to older adults with normal or near-normal, hearing suggest that listening working memory span (LWMS) in quiet may be a more informative measure than reading working memory span (RWMS). Pichora-Fuller et al. (1995) measured RWMS and LWMS in a group of younger listeners with normal hearing (*n* = 16) and in a group of older listeners with normal hearing through 3000 Hz (*n* = 16). The two tests followed the same protocol for determining working memory span. The reading measure used the same sentences as had been used in earlier studies by Daneman and Carpenter (1980, 1983). The listening measure used sentences from the Revised-Speech in Noise test (R-SPIN; Bilger, 1984) presented in quiet. Their results showed a significant correlation between the reading and LWMS measures for both the younger (*r* = 0.56) and older (*r* = 0.71) listener groups. Although an age-related difference in RWMS often is found (see Bopp and Verhaeghen, 2005 for a meta-analysis), in the study of Pichora-Fuller et al. (1995), both age groups had equivalent performance on the RWMS measure, perhaps because those in the older group were cognitively high-performing, well-educated, healthy older adults. Notably, despite the equivalent performance of the two age groups on the RWMS measure, younger adults had larger (better) LWMSs than the older adults and the older group performed worse on the LWMS test than on the RWMS test. The authors attributed this pattern of findings to age-related differences in supra-threshold auditory processing rather than to general modality-independent age-related differences in cognition, consistent with the domain-specific view of working memory. Similar results were found in a more recent study (Baldwin and Ash, 2011) in which RWMS and LWMS (in quiet) and speech-recognition threshold in quiet were tested in a group of younger (*n* = 80) and older (*n* = 26) adults with normal audiometric pure-tone thresholds through 8000 Hz. Specifically, the RWMS scores of the two age groups were similar, but the LWMS and speech recognition threshold results were significantly poorer for the older listeners compared to the younger listeners. A Pearson *r* correlation between RWMS and LWMS was not reported; however, a regression analysis showed that speech recognition thresholds, but not RWMS, predicted LWMS performance in older listeners, but not younger listeners. These studies comparing younger and older adults with normal or near-normal hearing (Pichora-Fuller et al., 1995; Baldwin and Ash, 2011) suggest that measuring LWMS may reveal age-related inter-individual differences relevant to listening performance on speech tests that are not revealed by measuring RWMS.

Another reason some studies may have been more successful than others in finding an association between measures of working memory and speech recognition is the selection of speech materials. Most studies have used various sentence-level materials in various listening conditions (e.g., quiet, noise, aided, etc.; see Akeroyd, 2008 and Besser et al., 2013 for reviews). Other studies have used phoneme or word-based materials (e.g., Akeroyd, 2008 for review; also see Humes and Floyd, 2005; Cervera et al., 2009; Baldwin and Ash, 2011; Smith et al., under review). A few studies have investigated associations between working memory and a range of speech materials in the same participants. For example, Humes and Floyd (2005) examined the associations among working memory (measured using a Simon-Says memory game (Pisoni and Cleary, 2004), presented in an auditory-only, visual-only, or auditory-visual condition) and two speech measures, a nonsense syllable test (City University of New York Nonsense Syllable Test [CUNY NST], Levitt and Resnick, 1978) and an open-set sentence recognition task (Connected Speech Test [CST], Cox et al., 1988), presented in unaided and aided conditions, in younger listeners with normal hearing (*n* = 12) and older listeners with hearing loss (*n* = 24; correlations were based on data for 22 of the 24 older listeners with hearing loss). Regardless of the modality, the Simon-Says task was not correlated with either speech measure in either condition in this study. In contrast, Cervera et al. (2009) did report significant correlations; specifically, they examined associations between two memory tests (serial recall and digit ordering) and two speech tests, a vowel-consonant-vowel (VCV) nonsense syllable repetition test (in quiet and in noise) and an open-set sentence recognition test (normal and fast speech rate) in 28 younger adult listeners with normal hearing and 27 older participants (mean age = 60 years) with mild, high-frequency hearing loss. The results showed that memory measures did not correlate significantly with the VCV materials, but a significant correlation emerged for both memory measures and fast-rate sentence recognition. These two studies illustrate the range of memory tests and speech materials used in listeners with and without hearing loss and across age groups (see also the reviews by Akeroyd, 2008; Besser et al., 2013).

In summary, a number of working memory and speech measures have been used to examine associations between working memory and speech understanding in adults with and without hearing loss. Discrepancies in findings may be attributable to the participants, the materials and the tasks used across the studies. We aimed to explore the associations between verbal working memory measures presented in the visual and auditory modalities and to determine if there would be modality-specific associations depending on the linguistic level of the materials (words, sentences, discourse), the nature of the task (simple repetition vs. comprehension) used to test speech understanding, and the age and hearing loss of the listener group. We hypothesized that there would be a significant correlation between LWMS and RWMS for all three groups, but that LWMS would be more strongly correlated than RWMS with speech measures, especially in the older listeners with hearing loss, when more linguistically complex materials were used and for the task involving comprehension rather than simple repetition of the speech materials.

## Materials and Methods

### Participants

**FIGURE 1:**
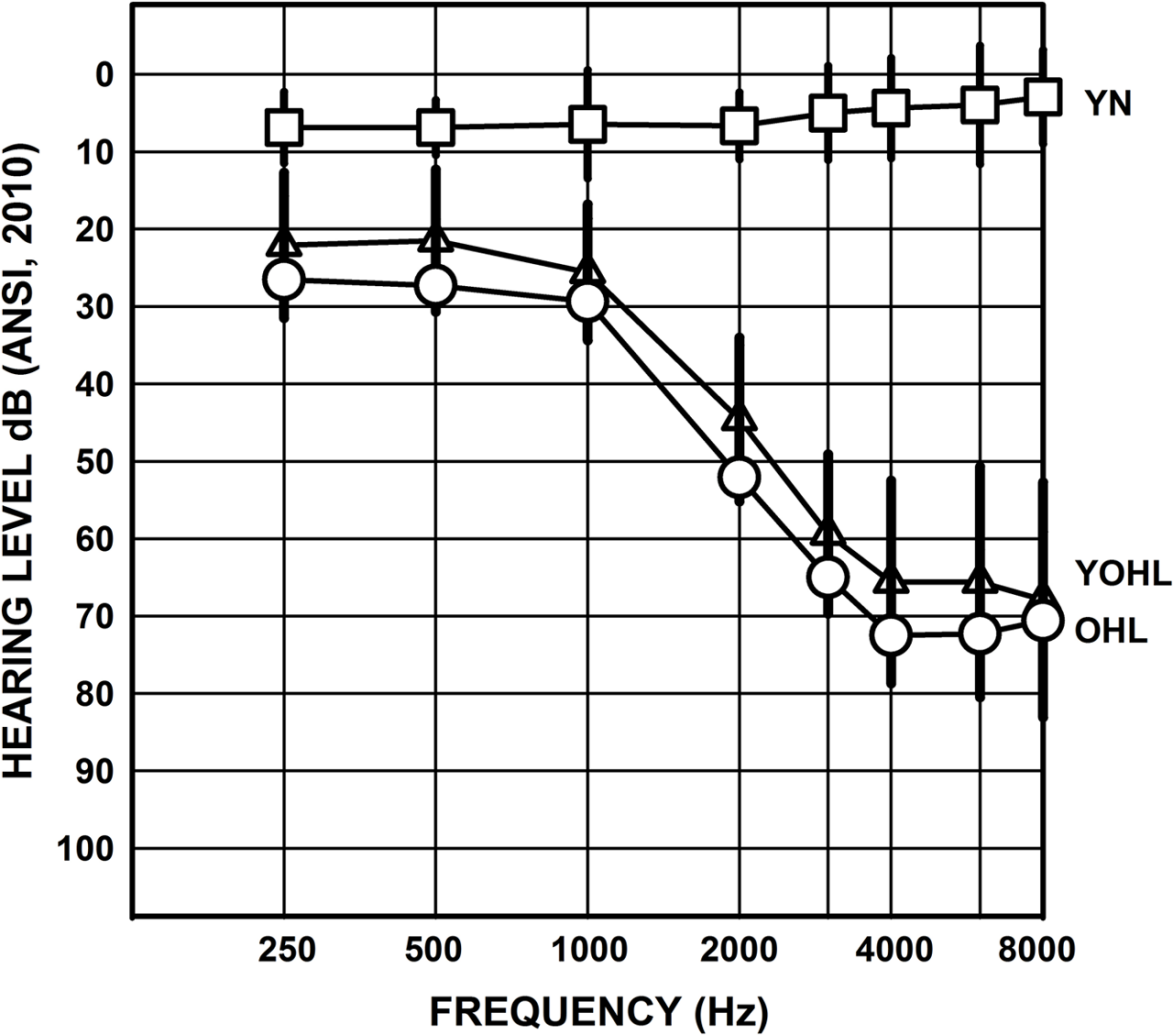
**Mean audiogram of the test ear for the younger listeners with normal hearing (YN; squares), young-old listeners with hearing loss (YOHL; triangles), and older listeners with hearing loss (OHL; circles).** The error bars represent one standard deviation.

Three listener groups participated (*n* = 24 per group)^1^. One group consisted of younger adults with normal hearing (YN; mean age = 23.5 years, *SD* = 2.8, range = 19–29; 7 male) who were recruited from the Johnson City, Tennessee community. The other two groups were older adults with hearing loss and were Veterans recruited from the Mountain Home, Tennessee Veterans Affairs (VAs) Medical Center Audiology clinic. The ‘young–old’ group (YOHL) had a mean age of 66.3 years (*SD* = 2.0, range = 63–69; 24 male), and the ‘older’ group (OHL) had a mean age of 74.3 years (*SD* = 3.2, range = 70–80; 24 male). A one-way analysis of variance (ANOVA) confirmed a significant difference in age among the three listener groups, *F*(2,71) = 2416.4, *p* < 0.001. The average education level was 15.3 years (*SD* = 2.1, range = 12–20) for the YN listeners, 14.9 years (*SD* = 2.7, range = 12–20) for the YOHL listeners, and 13.9 years (*SD* = 2.5, range = 8–18) for the OHL listeners; a one-way ANOVA indicated no significant group difference in education level (*p* > 0.05). **Figure 1** illustrates the average audiogram of the test ear of the three listener groups (right ear of even-numbered participants and left ear of odd-numbered participants). A repeated-measures ANOVA for audiometric thresholds across frequency of the test ear (within-subjects factor) with hearing loss groups (YOHL and OHL) as between-subjects factors, revealed no significant main effects of group, nor was there a frequency by group interaction (*p* > 0.05), suggesting similar test-ear audiograms for the YOHL and OHL groups.

The inclusion criteria were as follows: ability to speak American English; adequate vision and ability for reading verified by reading aloud a few sentences from the informed consent document; ≥50% correct word recognition accuracy in quiet to avoid floor effects with the test materials; >21/30 on the Montreal Cognitive Assessment to rule out dementia (Nasreddine et al., 2005); and no comorbid health condition (e.g., conductive hearing loss, substance abuse, blindness, mental health disorder, etc.) that potentially would interfere with the study procedures as determined by an interview (younger adults) or medical records review (older adults). Although tinnitus is a potential comorbid condition that may interfere with working memory (e.g., Rossiter et al., 2006), a positive history of tinnitus was not used as an exclusionary criterion^2^.

### Materials

A battery of five memory measures (three auditory and two visual) and six auditory measures of speech understanding were administered to each participant. These measures were chosen because of their availability and prior use in research and clinic applications. The memory measures included free recall and working memory presented in both the auditory and visual domains. The tests of speech understanding used a continuum of materials that varied in linguistic complexity (word, sentence, or discourse level materials) and tasks (simple repetition or comprehension). All auditory test materials were pre-recorded and most were spoken by the same talker (VA female speaker #2) drawn from a corpus of materials recorded by Wilson et al. (2008).

### Memory Tests

#### Reading Span (RS; Daneman and Carpenter, 1980)

The reading span test is a verbal working memory test administered in the visual domain using text. A total of 100 sentences are presented in five setsizes (2, 3, 4, 5, or 6 sentences per set) with five trials at each setsize. Thus, there are five 2-sentence trials (10 sentences); five 3-sentence trials (15 sentences); five 4-sentence trials (20 sentences), five 5-sentence trials (25 sentences), and five 6-sentence trials (30 sentences). The participant sees one sentence at a time (text via power point) and is asked to (1) read the sentence aloud, (2) make a judgment about whether or not each sentence makes sense (which serves to induce semantic processing of the entire sentence), and (3) at the end of a trial when prompted with a blank blue screen, the participant recalls the final word from each sentence in the trial in the order in which they were presented. The RS test was scored in terms of span size or the largest setsize for which the participant correctly recalls three out of five trials; however, partial credit is given for up to two out of three correctly recalled trials in the next highest set size.

#### Word Auditory Recognition and Recall Measure (WARRM; Smith et al., under review)

The WARRM is an auditory working memory measure. The general procedures of the WARRM follow the RS test paradigm, but audio-recorded (VA female speaker #2) monosyllabic words following a standard carrier phrase “*You will cite* … ” are used as the target items. As in the RS test paradigm, in the WARRM, there are 100 targets presented across five setsizes (2, 3, 4, 5, and 6 per set) with five trials being tested for each setsize. The participant is presented one item at a time and is asked to (1) repeat aloud the target word, (2) make a judgment about whether the first letter of the word is from the first half (A-M) or the second half (N-Z) of the alphabet (which serves to induce further processing of the word to be recalled), and (3) the participant recalls all of the target words in the trial in the order in which they were presented when prompted with 500-Hz, 500-ms tone at the end of a trial. The WARRM yields two scores, a word recognition accuracy score (percent correct), which served as one of the six speech measures in the current study (also described below), and a working memory span score.

#### Visual Free Recall (VFR; Adapted from Rabbitt, 1968, 1991)

This test uses a list of 15 words with each word presented individually on a plain white power-point slide with a 1-s inter-stimulus interval (ISI). After the series of words are presented, a yellow slide with the word ‘RECALL’ in black is used as the recall prompt. The participants are asked to write down as many words as they can recall from the list on a score sheet in 3 min. The test is scored by summing the number of correctly recalled words.

#### Auditory Free Recall (AFR; Adapted from Rabbitt, 1968, 1991; Park et al., 1996)

Analogous to the VFR test, a list has 15 audio-recorded (using VA female speaker #2) monosyllabic words presented individually with a 2-s ISI between words. Following the series of words, a 500-Hz, 500-ms prompting tone is presented to cue recall. There are no common words between the AFR and VFR measures.

#### Digit Span (DS)

A modified audio version of the Wechsler Adult Intelligence Scale (fourth edition, WAIS-IV, Wechsler, 2008) digit span (DS) subtest was used. Typically, the test is administered in a face-to-face interview format in which the examiner presents trials by live voice. A trial consists of a series of single digits spoken at a rate of one per second. The number of digits per trial increases during the test, with two trials for each span size, starting with a 2-DS size and terminating with a 9-DS size. Rather than the typical live voice test presentation method, to ensure a more standardized method of administration (e.g., consistent ISI, talker, and presentation level), the test was modified by using a series of monosyllabic digits (0 and 7 were replaced with monosyllabic digits) recorded by VA female talker #2, followed by a 500-Hz, 500-ms prompting tone. Otherwise, the general procedures of the DS test were maintained for the digit span forward (DSF), digit span backward (DSB), and digit span sequencing (DSS) subtests. For all subtests, the listener is presented with a series of digits, presented one at a time with a 1-s ISI, followed by the prompting tone. The response required from the listener varies with each subtest in that the listener is asked to recall the digits in the order in which they were presented (DSF), in the reverse order in which they were presented (DSB), or in the ascending numerical order in which they were presented (DSS). The subtests are scored by summing the number of correctly recalled trials.

### Speech Understanding Tests

#### Word Recognition in Quiet (from the WARRM)

An overall percent correct word recognition score across the 100 WARRM test items was calculated. This score served to determine word-recognition abilities in quiet for the same items for which recall also was tested (see above).

#### Words-In-Noise Test with VA Female Speaker #2 (WIN#2)

The original Words-In-Noise test (Wilson, 2003; Wilson et al., 2003) has two, 35-word lists presented in a six-talker background. The words are from the Northwestern University Auditory Test No. 6 (NU-6, Tillman and Carhart, 1966). For each list (List 1 and List 2), five words are presented at seven SNRs from 24- to 0-dB in 4-dB decrements. The WIN#2 test was modified by replacing the original NU-6 words with the same words spoken by VA female speaker #2 with the carrier phrase “*You will cite*” instead of the original “*Say the word*” carrier phrase. In the current study, the WIN#2 is scored by calculating the 50% point threshold (dB S/N) using the Spearman-Kärber equation and averaged across both lists (Finney, 1952; Wilson et al., 1973).

#### Multi-Signal-to-Noise Ratio Revised Speech in Noise Test (Multi-SNR R-SPIN; Wilson et al., 2012)

A modified version of the Revised Speech in Noise Test (R-SPIN; Bilger, 1984) was used. In this version, two 50-sentence lists containing R-SPIN sentences (from Lists 3 and 4, original male talker) were distributed across 10 signal-to-noise ratios (SNR, S/N) from 23- to 4-dB in 3-dB decrements, with five sentences at each SNR. Across the two lists and at each SNR, five low-probability (LP) and the corresponding five high-probability (HP) sentences were used. The listener is asked to repeat aloud the final word in each sentence. The test is scored by calculating separate 50% points (dB S/N via the Spearman-Kärber equation) for the sentence-final target words in the LP and HP sentences across the list pair, and there also is a linguistic context score (difference in 50%-point between HP and LP scores).

#### Quick Speech-in-Noise Test (QuickSIN; Killion et al., 2004)

Lists 1 and 2, along with a practice List A, of the QuickSIN were used (Etymotic Research, 2001). Each QuickSIN list consists of six Institute of Electrical, and Electronics Engineers (1969) sentences that are presented in a multi-talker background. One sentence is presented at each of 6 SNRs that range from 25- to 0-dB in 5-dB decrements. Each sentence is scored based on correct recognition of five keywords (e.g., A white silk jacket goes with any shoes.). In the current study, this test was scored in terms of the 50%-point (Spearman-Kärber) and an overall QuickSIN score was calculated by averaging the scores across Lists 1 and 2.

#### Veterans Affairs Sentence Test (VAST; Bell and Wilson, 2001)

The VAST sentences are constructed based on the Neighborhood Activation Model (Luce, 1986). Briefly, the monosyllabic words selected for the sentences are based on four lexical categories including: (1) sparse, or words that are unique or have few similar “neighbors,” (2) dense, or words with many phonetic similarities with other words (i.e., many lexical neighbors), (3) low use, or words that are infrequently used in spoken language, and (4) high use, or words that are frequently used on spoken language. Using these categories, four combinations of sentences types based on word frequency (either low or high use) and neighborhood similarity (either sparse or dense) were used to construct the VAST sentence lists, which included (1) low use, sparse (LS), (2) low use, dense (LD), (3) high use, sparse (HS), and (4) high use, dense (HD). Each participant was administered one 20-item VAST list that consisted of items from each sentence type (LS, LD, HS, and HD). Each sentence contains three keywords, and accuracy is scored in percent correct for the keywords for each list (60 keywords per list).

#### Lectures, Interviews, and Spoken Narratives Test (LISN; Tye-Murray et al., 2008)

Three spoken narratives (about 3 min each) from this test were used; two test narratives (Narrative 6 about an individual’s college experience, male talker; Narrative 7 about a store fire, male talker) along with a practice narrative (Narrative 10 about a grocery store robbery, female talker). The narratives were spoken by different talkers in a natural, conversational style. Participants listened to each narrative in its entirety and answered six multiple-choice comprehension questions, each with four response choice alternatives (pen/paper format). These questions asked about three different aspects of listening comprehension including: (1) information (i.e., recalling a specific detail in the narrative), (2) integration (i.e., the listener’s ability to combine pieces of information), and (3) inferences (i.e., the listener’s ability to infer implications from the narrative). There are two questions for each aspect of listening comprehension. An overall listening comprehension score along with a score for each question type was calculated for each list and averaged across lists as a percent correct score.

### Procedures

The study was approved by the local research ethics committees (East Tennessee State University/VA Institutional Review Board and VA Research and Development Committee). All groups provided informed consent prior to testing. After consenting, a pure-tone audiogram was obtained for the test ear (odd-numbered participants received testing in the left ear and even-numbered participants in the right ear) for octave frequencies of 250–8000 Hz and the inter-octave frequencies of 3000 and 6000 Hz (American National Standards Institute [ANSI], 2010). The YOHL and OHL listeners were administered a 25-word NU-6 list to ensure they had adequate word recognition abilities (>50%) in the test ear to complete the protocol. All groups received the MoCA to ensure that no participant had a positive screen for dementia).

All visually presented materials were administered in a quiet lab space while the participant was seated at a table. The RS and VFR tests were administered using a computer (Dell, Model Optiplex 780) and a 15-inch computer screen (Dell 1908FP). Participants wore their habitual corrective lenses during testing if needed for reading. The YOHL and OHL listeners either wore their hearing aids (if they owned them) or a pocket talker during MoCA administration (Dupuis et al., 2015) and when test instructions were given to ensure they could hear the instructions optimally.

All audio-record materials were presented from a compact disc (CD) that was calibrated and then played through a CD player (Sony, Model CDP-CE375) routed through an audiometer (Grason-Stadler, Model 61) to an insert earphone (Etymōtic, Model ER-3A) while the participant was seated in a double-walled sound-attenuating booth. The NU-6 words, WARRM, modified DS, AFR, VAST, and LISN were all presented in quiet at presentation levels of 62 dB HL for YN listeners, 72 dB HL for YOHL and OHL with pure-tone averages (PTA at 500, 1000, and 2000 Hz) < 40 dB HL, and 82 dB HL for YOHL and OHL with PTAs 40–60 dB HL. The WIN#2 and multi-SNR R-SPIN were presented at 80 dB SPL (equivalent to 62 dB HL) for listeners with PTAs < 40 dB HL, and 90 dB SPL (equivalent to 72 dB HL) for listeners with PTAs 40-60 dB HL, with the levels used for the WIN#2 and multi-SNR R-SPIN based on the level of the noise, which was held constant while the level of the speech was varied to yield the range of SNRs tested. The presentation level of the QuickSIN lists followed the administration manual and were presented at 70 dB HL for participants with PTAs ≤ 45 dB HL and at a dial level that was “loud, but OK” for participants with PTAs ≥ 50 dB HL.

All listener groups completed the testing in two sessions. The tests for the experimental protocol were sequenced so that the tests were balanced across sessions to avoid fatigue and order effects. Session One lasted ~80–90 min for each listener group. After consenting and testing for inclusion/exclusion criteria in Session One, all groups then were administered the RS and WARRM tests; the order of the tests was counterbalanced. The RS and the WARRM were grouped together because of similarities in their testing procedures. A 10-min break was required between these two working memory tests for the older groups, whose testing for Session One ended after the RS and WARRM testing was completed. For the younger listeners, there was a 10-min break required after the RS and WARRM testing, followed by the WIN#2 and the QuickSIN tests, with these tests counterbalanced across participants. Session Two lasted ~60 min for the YN listeners and 90 min for older listeners. For Session Two, the session was divided into two halves, with one half of the session focusing on speech understanding testing and the other half of the session focusing on memory testing. The session halves were counterbalanced across participants and a 10-min break was required between the halves. For all groups, the memory testing half of Session Two included the DS, AFR and VFR measures. The DS and the VFR tests were administered in a counterbalanced order, either first or last, with the AFR always being administered between them. The AFR and VFR tests were administered consecutively because of the similarities in the test procedure. The VFR test was either administered first or last in the session to minimize changes in test locations (either the sound booth or computer location) within the session half.

For the younger listeners, in the speech understanding testing half of Session Two, the LISN and VAST tests were counterbalanced, with the multi-SNR R-SPIN test always administered in between them because it was considered to be less demanding than the LISN and VAST tests. For the older listeners, in the speech understanding testing half of the Session Two, participants were administered the LISN, VAST, QuickSIN, WIN#2, and multi-SNR R-SPIN tests; the LISN or VAST were administered first or third (counterbalanced across participants) and the QuickSIN, WIN#2 or multi-SNR R-SPIN test were randomly assigned as the second, fourth, or fifth tests. The rationale for this ordering of tests was to administer a more demanding test followed by one that was less demanding to avoid fatigue for the older listeners. Because multiple lists were administered for a given speech understanding test, the list order of the speech tests also was counterbalanced to avoid order/list effects. For the QuickSIN and LISN tests only, a practice list was administered prior to the experimental lists. The four VAST lists were assigned randomly to each participant. The participants were encouraged to take additional breaks during testing as needed and were remunerated $20 per hour.

## Results

Several measures were administered to three groups of participants (YN, YOHL, OHL) to assess their cognitive and speech understanding abilities. Descriptive results and group differences on each measure were calculated. Correlational analyses were performed to examine the associations between reading and LWMS. An ANOVA was conducted to examine the effect of test modality on working memory span. Finally, the contributions of memory to performance on various speech understanding measures were evaluated using correlational analyses. All data were analyzed with statistical software (International Business Machines Statistical Package for the Social Sciences, Version 22.0) and all analyses (ANOVAs and *post hoc* analyses) were adjusted (Bonferroni) to account for multiple comparisons.

In **Table 1**, the mean results for seven memory measures are listed for each group. For each variable, a separate one-way ANOVA was conducted to evaluate group differences and those results also are presented in the table. The ANOVAs revealed significant differences among the results for the groups on all memory measures. In all cases where there was a significant group difference, *post hoc* analyses showed that the younger listeners performed best, and the two groups of older listeners had similar performance that was significantly poorer than that of the younger listeners.

**Table 1:** The mean performance (and one standard deviation) on the seven memory measures by the three listener groups.

	YN		YOHL		OHL				
	*M*	*SD*	*M*	*SD*	*M*	*SD*	*F*	*df*	*p*
Reading span	2.5	0.9	1.9	0.3	1.9	0.4	8.4	2, 69	**0.001**
WARRM span	4.4	1.1	3.0	0.6	2.9	0.8	23.7	2, 69	**0.000**
Visual free recall	8.0	1.7	4.8	1.5	4.2	1.7	37.1	2, 69	**0.000**
Auditory free recall	8.3	2.2	4.8	1.8	3.5	1.4	45.0	2, 69	**0.000**
Digit span									
Forward	10.3	2.2	9.1	1.8	9.1	2.4	2.4	2, 69	0.102
Backward	8.8	1.9	7.7	1.6	7.3	2.5	3.2	2, 69	0.047
Sequencing	10.1	1.8	9.4	1.5	8.0	1.9	3.2	2, 69	**0.000**

*The results from separate one-way analyses of variances also are listed. YN, younger listeners with normal hearing; YOHL, young–old listeners with hearing loss; OHL, older listeners with hearing loss; WARRM, Word Auditory Recognition and Recall Measure. Shown in bold are *p* values < 0.007 which were considered to be significant after Bonferroni corrections were applied.*

For each listener group, correlations were computed to explore the associations among the memory measures (only *ps* < 0.007 were considered to be significant). For the listeners in the YN group, Pearson *r* correlations were significant between AFR and DSB (*r* = 0.61, *p* = 0.002) and between AFR and DSS (*r* = 0.59, *p* = 0.002). No significant correlations were found among the memory measures for the YOHL listeners. For the OHL listeners, Pearson *r* correlations were significant between WARRM span and DSS (*r* = 0.55, *p* = 0.006); WARRM span and VFR (*r* = 0.55, *p* = 0.005); and DSB and DSF (*r* = 0.63, *p* = 0.001). Correlations between the RS and WARRM span will be presented later as they address a distinct aim of the study.

**Table 2** lists the mean performance for each listener group from the six speech tests and subtests if applicable. The results of the one-way ANOVAs to evaluate group differences on the speech measures also are presented in **Table 2** (*p* values < 0.003 were considered to be significant). The ANOVAs revealed a significant difference among groups for each speech understanding measure except for the measures from the LISN test and the Use of Context measure from the multi-SNR R-SPIN test. For WARRM recognition, the Low Probability measure from the multi-SNR R-SPIN test, and the HS, HD and LS measures from the VAST test, the younger group performed the best, followed by the two older groups who performed similarly. A different pattern emerged for the WIN#2, the High Probability measure from the multi-SNR R-SPIN test, the QuickSIN, and the LD measure from the VAST test, with all three groups performing significantly differently from each other; the YN group performed best, followed by the YOHL group, with the OHL group performing worst.

**Table 2:** The mean performance (and one standard deviation) on the speech understanding measures by the three listener groups.

	YN		YOHL		OHL				
	*M*	*SD*	*M*	*SD*	*M*	*SD*	*F*	*df*	*p*
WARRM recognition (%)	*99.0*	*1.1*	79.7	9.2	76.2	13.5	40.5	2, 69	**0.000**
WIN#2 (dB S/N)	*3.0*	*1.0*	*14.0*	*3.3*	*16.0*	*3.3*	155.4	2, 69	**0.000**
multi-SNR R-SPIN									
Low-Probability (dB S/N)	*5.4*	*1.4*	9.7	2.8	10.9	3.1	30.5	2, 69	**0.000**
High-Probability (dB S/N)	*1.5*	*1.2*	*4.2*	*2.0*	*5.6*	*2.3*	29.6	2, 69	**0.000**
Use of Context (dB)	3.9	1.4	5.5	1.8	5.2	1.9	6.3	2, 69	0.003
QuickSIN (dB S/N)	*3.2*	*1.3*	*8.9*	*3.5*	*11.2*	*3.8*	43.8	2, 69	**0.000**
VAST (%)									
Low Use, Sparse	*98.5*	*2.2*	90.9	5.4	87.4	8.2	22.5	2, 69	**0.000**
Low Use, Dense	*98.4*	*2.1*	*92.4*	*4.9*	*88.0*	*7.9*	*21.9*	2, 69	**0.000**
High Use, Sparse	*99.4*	*1.2*	96.0	3.8	94.1	4.5	14.8	2, 69	**0.000**
High Use, Dense	*99.2*	*1.0*	93.5	3.9	92.8	5.3	20.2	2, 69	**0.000**
LISN (%)									
Overall	76.7	14.5	72.9	15.4	62.8	19.0	4.6	2, 69	0.014
Information	80.2	18.0	74.0	21.5	61.5	23.3	4.9	2, 69	0.010
Integration	71.9	17.0	78.1	17.0	75.0	23.3	0.6	2, 69	0.537
Inferences	78.1	25.9	66.7	21.7	52.1	27.5	6.5	2, 69	0.003

*The results from separate one-way analyses of variances also are listed. YN, younger listeners with normal hearing; YOHL, young–old listeners with hearing loss; OHL, older listeners with hearing loss. WARRM, Word Auditory Recognition and Recall Measure (recognition score); WIN#2, Words-In-Noise Test Number 2; QuickSIN, Quick Speech in Noise test; multi-SNR R-SPIN, multi signal-to-noise ratio Revised Speech in Noise test; VAST, Veterans Affairs Sentence Test; LISN, Lectures, Interviews and Spoken Narratives test. Only *p* values < 0.003 were considered significant and are bolded. Italics are used to indicate the two patterns of results, either that the results of the younger group differed from those of the two older groups which did not differ from each other (only results of the younger group are italicized) or that all three groups differed significantly from each other (results for all three groups are italicized).*

For each listener group, correlations were computed to explore the associations among the speech understanding measures (only *ps* < 0.003 were considered to be significant). The significant correlations for the YOHL (below the diagonal) and the OHL (above the diagonal) listeners are listed in **Table 3**. Note the WARRM in **Table 3** refers to the word recognition score. The correlations for both hearing loss listener groups were mostly non-significant, with moderate to strong correlations for those correlations that were significant.

**Table 3:** The Pearson *r* correlations among the speech measures for the YOHL group (below the diagonal) and for the OHL group (above the diagonal).

	1	2	3	4	5	6	7	8	9	10	11	12	13	14
(1) WARRM		-0.67	.	.	.	-0.76	0.83	0.66	.	.	.	.	.	.
(2) WIN#2	-0.77		0.60	0.69	.	0.68	-0.67	.	.	.	.		.	
(3) LP	.	0.73		0.79	0.69	.	.	.	.	.	.	.	.	.
(4) HP	.	0.64	0.76		.	.	.	.	.	.	.	.	.	.
(5) Context	.	.	0.71	.		.	.	.	.	.	.		.	
(6) QuickSIN	-0.71	0.74	.		.		-0.63	.	.	-0.59	.		.	
(7) LS	0.67	.	.	.	.	-0.66		0.77	.	.	.	.	.	.
(8) LD	.	.	.		.	-0.67	.		0.59	0.74	.		.	
(9) HS	.	-0.61	.		.	.	0.67	.		0.59	.		.	
(10) HD	0.68	.	.	.	.	.	0.65	.	.		.	.	.	.
(11) LISN	.	.	.	.	.	.	.	.	.	.		0.72	0.80	0.80
(12) Info.	.	.	.	.	.	.	.	.	.		0.87		.	.
(13) Integ.	.	.	.	.	.	.	.	.	.		0.65	.		.
(14) Infer.	.	.	.	.	.	.	.	.	.		0.76	.	.	

*YOHL, young–old listeners with hearing loss; OHL, older listeners with hearing loss. WARRM, Word Auditory Recognition and Recall Measure (recognition score); WIN#2, Words-In-Noise Test Number 2; LP, low probability multi signal-to-noise ratio Revised Speech in Noise test (multi-SNR R-SPIN); HP, high probability multi-SNR R-SPIN; Context, multi-SNR R-SPIN Use of Context; QuickSIN, Quick Speech in Noise test; LS, low usage, spare Veterans Affairs Sentence Test (VAST); LD, low usage, dense VAST; HS, high usage, sparse VAST; HD, high usage, dense VAST; LISN, Lectures, Interviews and Spoken Narratives Test overall score; Info., information score on LISN; Integ., integration score on LISN; and Infer., Inference score on LISN.*

For the YN listeners whose results are not listed in **Table 3**, significant Pearson *r* correlations were observed between the QuickSIN and the VAST LS (*r* = -0.66, *p <* 0.001). For the LISN test, the overall score was significantly correlated with the LISN information score (*r* = 0.86, *p <* 0.001) and LISN inference score (*r* = 0.78, *p <* 0.001). For the multi-SNR R-SPIN test, the Low Probability measure was significantly correlated with Use of Context measure (*r* = 0.64, *p <* 0.001). No other significant correlations among the speech measures for YN listeners were found.

**FIGURE 2:**
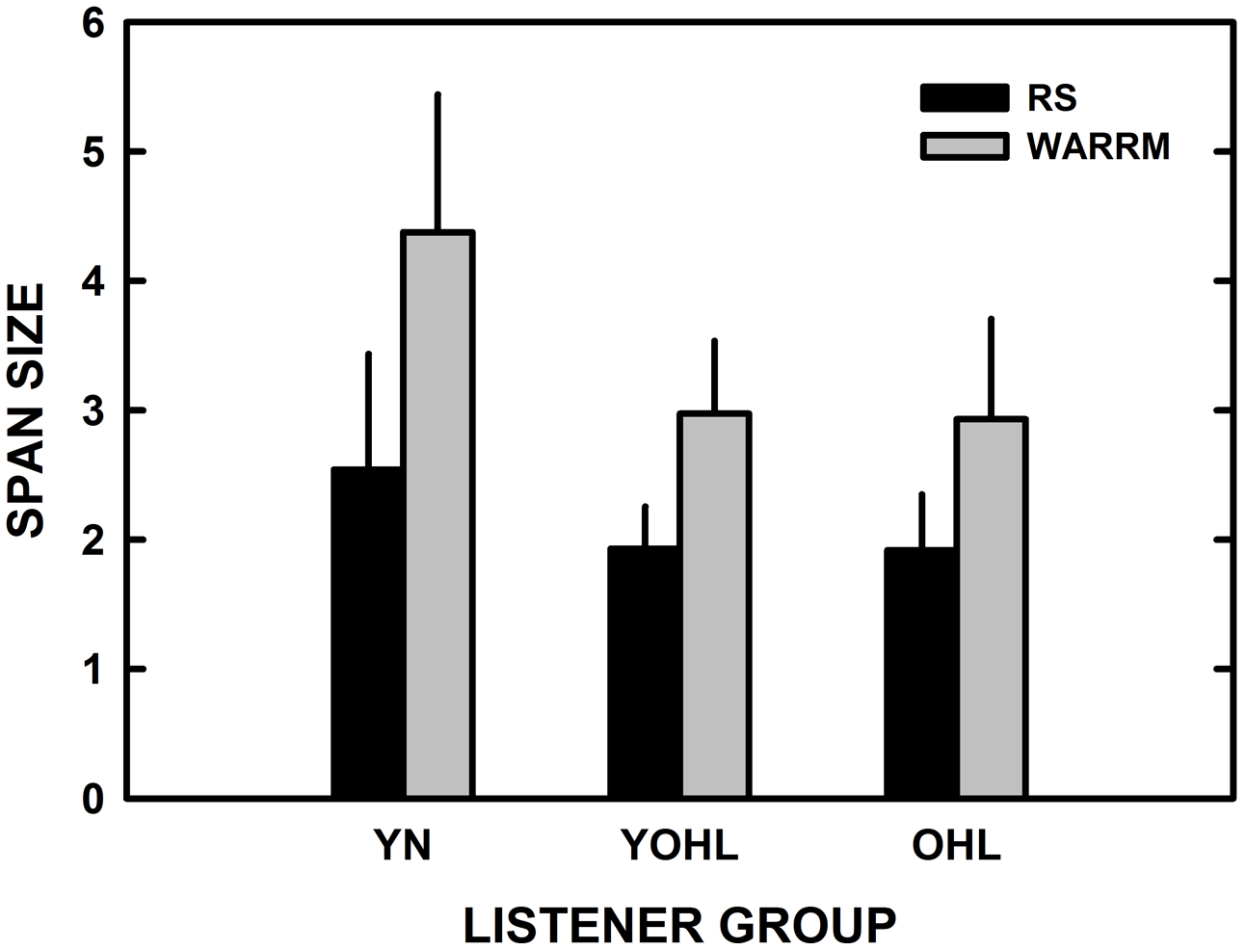
**The mean reading span (RS; black) and Word Auditory Recognition and Recall Measure (WARRM) span size scores (gray) are plotted as a function of listener group.** The error bars represent one standard deviation. YN, young–old listeners with hearing loss; YOHL, young–old listeners with hearing loss; and OHL, older listeners with hearing loss.

The results obtained for the RS (visual) and WARRM (auditory) working memory tests were compared to evaluate differences due to test modality. **Figure 2** illustrates the mean performance on the RS and WARRM tests for each listener group. A repeated measures ANOVA with group as the between-subjects variable (YN, YOHL, and OHL) was performed using span scores to compare test modalities (visual with the RS and auditory with the WARRM) as the within-subjects variable. The results showed a main effect of modality, *F*(1,69) = 172.5, *p* < 0.001, $\eta_{p}^{2}$ = 0.71, a main effect of group, *F*(2,69) = 23.9, *p* < 0.001, $\eta_{p}^{2}$ = 0.41, and a group by modality interaction, *F*(2,69) = 7.4, *p* < 0.001, $\eta_{p}^{2}$ = 0.18. *Post hoc* analyses showed that for the main effect of group (collapsed across RS and WARRM), the younger group performed best, followed by the two older groups, who had similar performance. For the main effect of modality (collapsed across group), performance was better on the WARRM span auditory test compared to the visual RS test. For the group by modality interaction, all groups performed better on the WARRM span (auditory) test relative to the RS (visual) test, but the difference between performances on these measures was larger for the younger listeners with normal hearing compared to the older listener groups who had similar differences in performance between the span measures.

**FIGURE 3:**
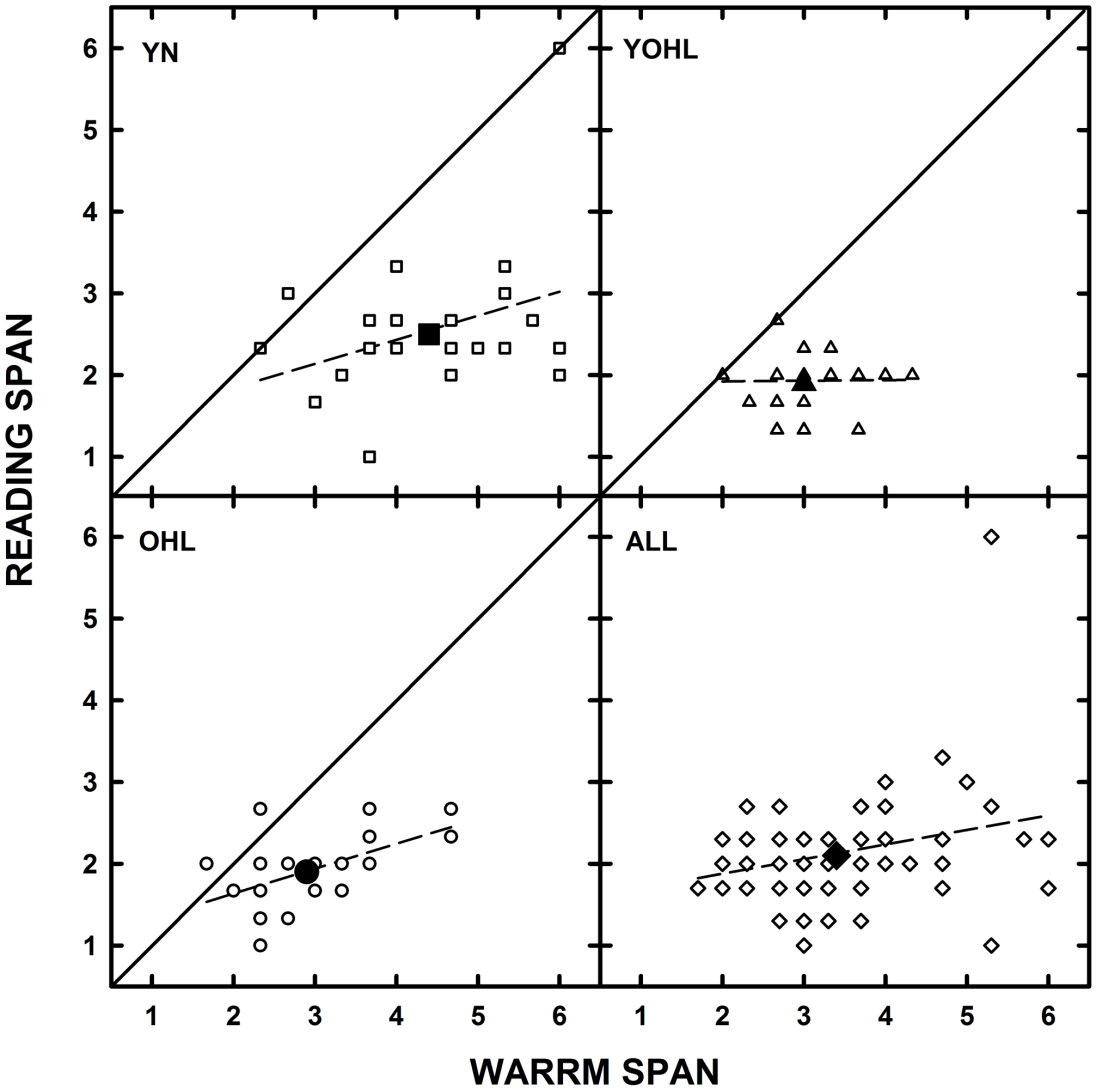
**The individual datum points (open symbols) for reading span are plotted as a function of Word Auditory Recognition and Recall Measure (WARRM) span scores for the younger listeners with normal hearing (YN; squares), young-old listeners with hearing loss (YOHL; triangles), older listeners with hearing loss (OHL; circles), and all participants in each panel respectively.** The large-filled symbols represent the group mean data. The solid line represents equal performance and the dashed line represents the linear regression through the datum points.

For each group separately and for all participants combined, Pearson *r* correlations were conducted to examine the associations between RS and WARRM span scores (see **Figure 3**). For all groups, the correlation was *r* = 0.52, *p* < 0.001 (significant at the 0.01 level, two-tailed). When correlations were computed for each group, the only significant correlation was for the OHL group (*r* = 0.55, *p* = 0.006).

**FIGURE 4:**
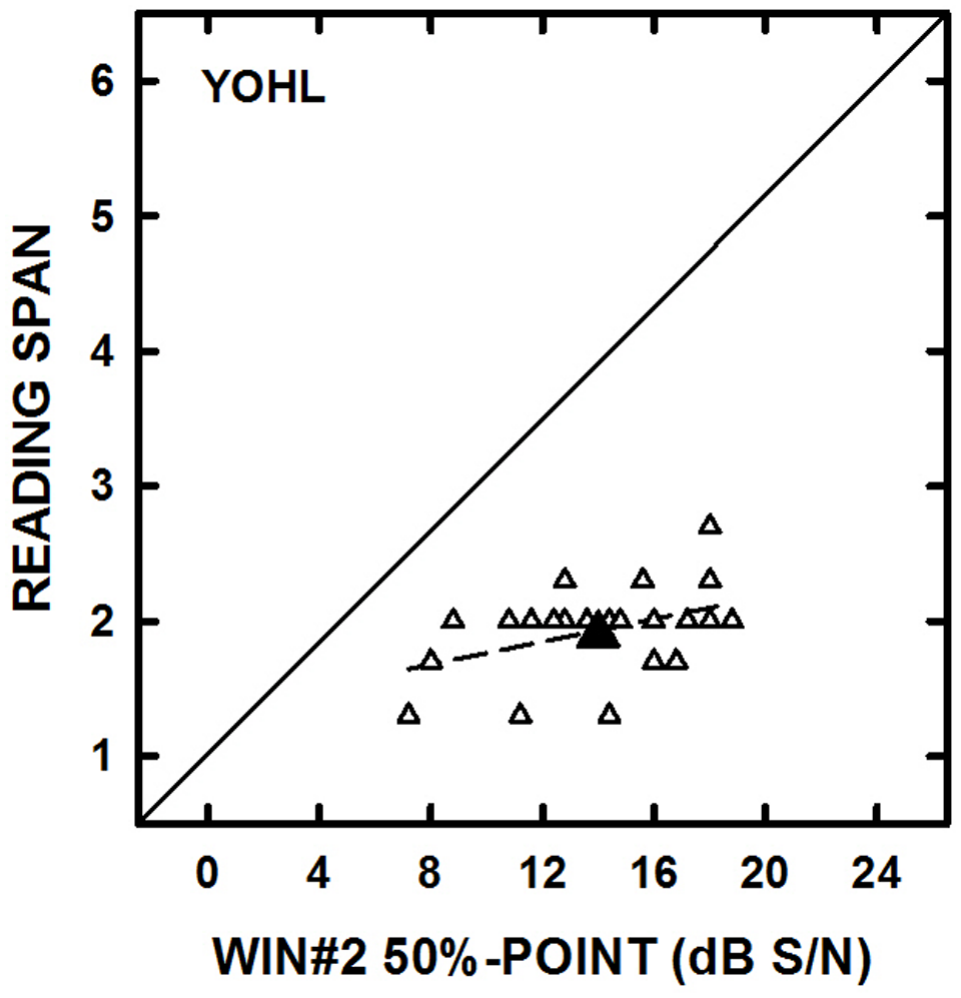
**The individual datum points (open symbols) for reading span are plotted as a function of performance on the Words-In-Noise #2 Test (WIN#2) for the young–old listeners with hearing loss (YOHL).** The large-filled symbols represent the group mean data. The solid line represents equal performance and the dashed line represents the linear regression through the datum points.

**FIGURE 5:**
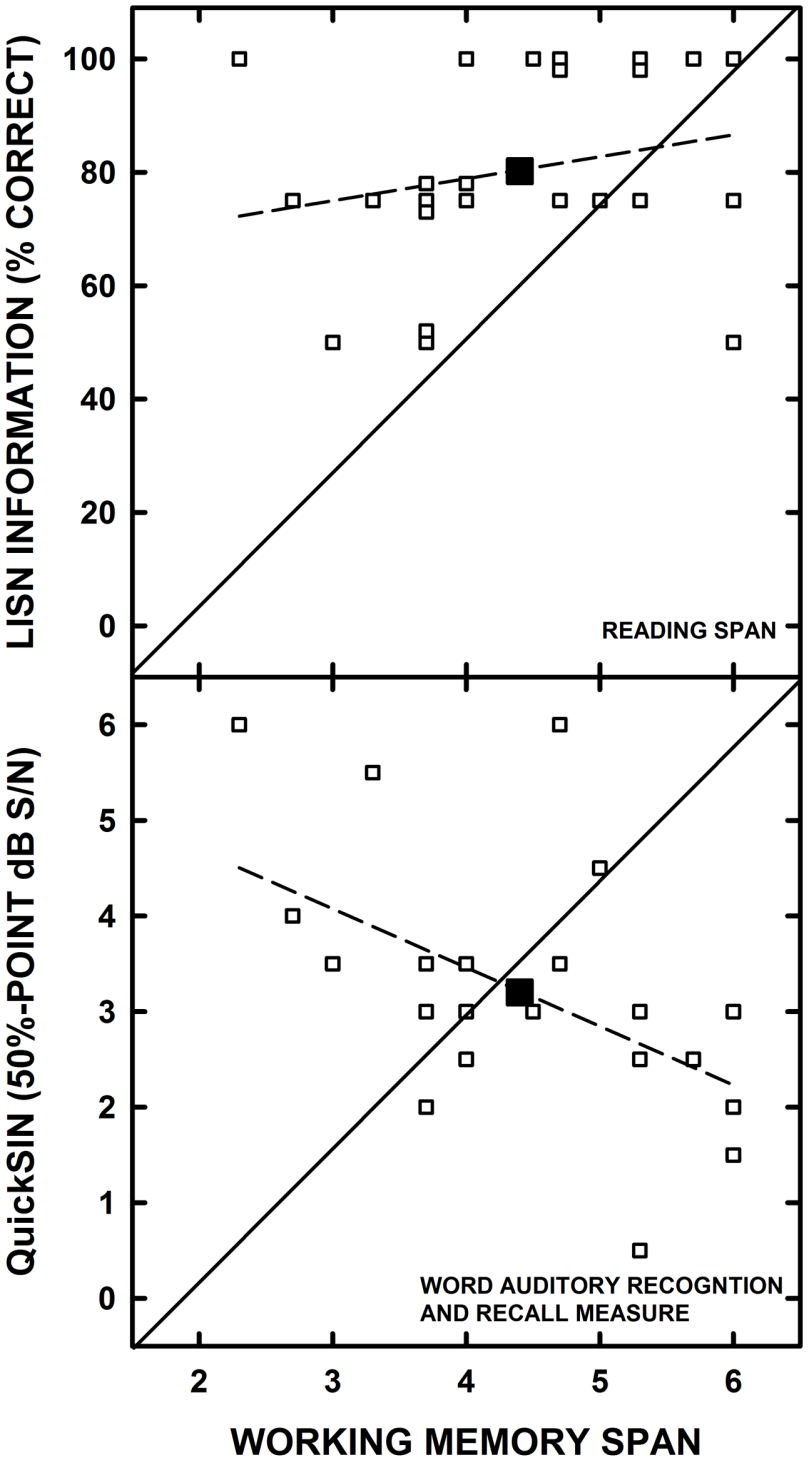
**The individual datum points (open symbols) for Lectures, Interviews and Spoken Narratives (LISN) Information scores are plotted as a function of Reading Span **(top)** and Quick Speech-in-Noise test scores are plotted as a function of Word Auditory Recognition and Recall Measure (WARRM) span scores are **(bottom)** for the younger listeners with normal hearing.** For graphical clarity, the data were jittered slightly to offset overlapping datum points. The large-filled symbols represent the group mean data. The solid line represents equal performance and the dashed line represents the linear regression through the datum points.

For each group, separate correlation analyses (controlling for high-frequency pure-tone average of 1000, 2000, and 4000 Hz) were conducted to examine the associations between the RS and WARRM span measures and each speech understanding measure. The only significant correlation found for the YOHL group was between the RS and WIN#2 scores (*r* = 0.49, *p* = 0.02; see **Figure 4**). For the YN listeners, WARRM span was significantly correlated with the QuickSIN (*r* = -0.48, *p* = 0.02) and RS was correlated with LISN information (*r* = 0.47, *p* = 0.02; see **Figure 5**). Aside from the few significant correlations, the general lack of significant correlations did not support our hypotheses that working memory would be correlated with results on tests of speech understanding and that the correlations would strengthen as the linguistic complexity of speech materials increased, particularly for OHL listeners. In fact, there were no significant correlations between working memory and speech understanding measures for the OHL listeners.

For the current study, we selected speech measures with the presumption that there would be increasing demands on working memory as linguistic complexity increased from words, to sentences, and then discourse. We expected that RS and WARRM would be significantly correlated with performance on tests of speech understanding, but that the strengths of those correlations would depend on the linguistic properties of the speech materials. In addition, we expected the strength of the correlations to be stronger for WARRM than RS depending on the auditory abilities of the participants. Because our hypotheses were not supported by the correlational analyses, we conducted a factor analysis to examine further the relations among the measures of memory and speech understanding. To this end, a principal components factor analysis using varimax rotation was conducted. Data from all participants (*n* = 72) were included. All speech understanding measures and memory measures, along with age and degree of hearing loss (determined by the pure-tone average [PTA] of 500, 1000, and 2000 Hz), were inputted into the analysis. The results revealed a five-factor solution that explained 76.9% of the variance (**Table 4** shows factor loading values > 0.60 for all five factors). The scree plot, however, suggested that the first three factors may be the most appropriate components to include in the solution. In general, as can be seen in the table, the majority of the speech understanding measures, along with age and PTA (which typically are correlated with speech measures), loaded on Factor 1. The majority of the memory measures loaded on Factor 2. The LISN (sub)tests loaded on Factor 3 and the Use of Context score from the multi-SNR R-SPIN loaded on Factor 4. The DSF loaded on Factor 5. These results suggest that there is a similarity amongst age, PTA and the speech understanding measures when the speech understanding task is simply to repeat words or sentences, whereas the speech understanding measures involving the comprehension of discourse or the use of semantic context are separate factors. Importantly, the majority of the memory measures were distinct from both kinds of speech understanding measures, and also the more basic and less cognitively demanding DSF memory measure.

**Table 4:** The factor loading values (sorted by strength) and the percent variance explained for each factor resulting from the factor analysis results are listed.

	Factor 1	Factor 2	Factor 3	Factor 4	Factor 5
WARRM Recognition	0.91				
VAST LS	0.86				
QuickSIN	-0.85				
WIN#2	-0.84				
VAST HS	0.83				
VAST LD	0.83				
VAST HD	0.81				
multi-SNR R-SPIN HP	-0.77				
Pure-tone average	-0.73				
Age	-0.70				
multi-SNR R-SPIN LP	-0.69				
AFR	0.64	0.60			
RS		0.76			
WARRM span		0.66			
VFR		0.64			
LISN overall			0.96		
LISN information			0.76		
LISN integration			0.73		
LISN inference			0.69		
multi-SNR R-SPIN context			0.89	
DSF					0.90
Percent variance	35.4	15.2	13.0	6.6	6.6

## Discussion

The main study aim was to examine the effect of presentation modality (auditory or visual) on verbal working memory measures in different listener groups. As expected, there was a significant effect of group, with the YN listeners outperforming the YOHL and OHL listeners on verbal working memory measures tested in both modalities. Previous studies have demonstrated such age effects on working memory measures, in particular, in studies using the reading span measure (e.g., see Bopp and Verhaeghen, 2005 for a meta-analysis). Little data exists for the newly developed WARRM measure; however, previous data comparing 48 YN listeners with normal hearing to 48 older listeners with normal to near-normal hearing (ONH) and 48 older listeners with hearing loss revealed significant differences in mean WARRM spans suggesting that age affects performance on this measure (4.7 vs. 3.9 for the YN and ONH groups, respectively) and that hearing loss also affects performance (3.9 and 3.6 for the ONH and the OHL groups, respectively (Smith et al., under review).

The current results indicate that, for all listener groups, WARRM span was significantly higher and more variable than RS. There are a number of possible explanations for the difference in span size between the WARRM and RS tests. First, working memory span measures have been shown to be sensitive to the complexity of linguistic processing required for comprehending sentences (Waters and Caplan, 1996). Both the RS and WARRM measures use sentence-length stimuli, but the RS stimuli are a set of unique sentences, whereas the WARRM stimuli are monosyllabic words following a standard carrier phrase. Thus, because the WARRM stimuli are simpler and require less linguistic processing compared to the RS sentences, it would be expected that participants should be able to store more WARRM target words than RS target words. Second, for the RS measure, participants were asked to read aloud each sentence as they progressed through the recall set, thereby reducing the opportunity to rehearse the previous final words in the trial. In contrast, for the WARRM measure, each target word was presented following the same carrier phrase (‘*You will cite*’) and only the target word was repeated. Thus, even though the ISI between individual words on the WARRM was short (3 s) and was intended to leave time only for repetition of the target word and the linguistic judgment task, participants may have had more opportunity to rehearse the target words in the ISI or during the carrier phrase. Third, serial recall can be affected by word length such that monosyllabic word sequences are recalled more accurately than are multi-syllable word sequences (e.g., Baddeley et al., 1975), possibly because of differences due to word length in rehearsal opportunity or forgetting during the recall response period (Baddeley, 2003). The final words to be recalled in the RS test included both monosyllabic and multi-syllabic words and the inclusion of multi-syllabic words may have resulted in more forgetting on the RS than in the WARRM test. In short, linguistic differences between the RS and WARRM stimuli may have differentially affected processing requirements, opportunities for rehearsal and propensity for forgetting, resulting in better performance in the WARRM span relative to the RS for all listener groups. It seems unlikely, however, that individual differences in linguistic abilities would have resulted in greater variability on the linguistically easier WARRM test compared to the more linguistically difficult RS test. Rather, less variability should have been observed on the easier WARRM test than on the harder RS test if linguistic processing were the explanation for inter-test differences.

A significant interaction between verbal working memory test modality and group was found. The interaction emerged because the difference between the two working memory measures was larger, almost twice as large for the YN listeners (1.9) relative to the two older listener groups (1.0 and 1.1, respectively; see **Table 1** and **Figure 2**). For the RS test, the differences in spans between the groups were small (by ~0.5 span size), but the pattern of differences between groups did demonstrate the typical age effects. For the WARRM, the effect of age also was observed; however, there were larger group differences on the WARRM test (by about 1.5 span units of difference between YN and YOHL/OHL groups) compared to the RS test. The YN listeners have normal pure-tone thresholds and presumably better auditory processing relative to the two older listener groups who have hearing loss. It is likely that the higher WARRM spans for the YN listeners could be attributed to their relative ease in hearing the WARRM stimuli compared to the older listeners with hearing loss. Accordingly, the difference between the two working memory measures within groups was largest for the YN compared to the other two groups, possibly reflecting differences in age and auditory processing abilities among the groups. It also seems reasonable that individual differences in auditory processing abilities might explain the greater variability observed in the results on the WARRM test than in the results on the RS test.

There was a significant moderate correlation (*r* = 0.55) between the RS and WARRM span measures for the OHL group only. Previous studies have found moderate correlations between LWMS and RWMS measures for younger (Pichora-Fuller et al., 1995; Besser et al., 2013), middle-age (Koelewijn et al., 2012), and older listeners with normal hearing (Pichora-Fuller et al., 1995). Although the current study did not demonstrate such correlations for YN and YOHL listeners, the results provide evidence that listening and reading span measures are moderately associated in older listeners with hearing loss. Furthermore, as can be seen in **Figure 3**, there is more variability in the individual datum points for WARRM span (abscissa) relative to RS (ordinate). Thus, the small range in performance on the RS likely contributed to a lack of a significant correlation between the measures for the YN and YOHL listeners. For researchers or clinicians interested in examining inter-individual differences in verbal working memory and how those differences relate to individual differences in speech understanding, given the greater range in performance on the WARRM test relative to the RS test, the WARRM may be a better metric to capture individual differences in verbal working memory across a range of listener groups.

The second aim of the present study was to examine the extent to which verbal working memory (RS or WARRM span) is associated with various measures of speech understanding for the different listener groups. Our hypothesis was that working memory would become more strongly correlated as the level of linguistic complexity of the materials increased (from word to sentence to discourse) and as the task shifted from simple repetition to comprehension. We also expected that WARRM span would be more strongly correlated than RS with measures of speech understanding, especially as linguistic complexity increased and especially for older adults with hearing loss. Contrary to our prediction, more significant correlations were found for YN listeners than the other groups, but the strength of the correlations did not change as a function of linguistic complexity or modality of the working memory measure. The observation of more significant correlations for the YN group may have arisen because their performance was not affected by hearing loss. Previous research has suggested that working memory emerges as a small, but significant factor explaining speech understanding, particularly speech-in-noise performance, only after audibility is accounted for, either by manipulation of the presentation level or through amplification, but that without correction for hearing loss the variance due to working memory is dominated by measures of hearing loss (Akeroyd, 2008; Houtgast and Festen, 2008; Humes et al., 2013). In the current study, the level of presentation of the speech stimuli was selected based on the hearing level of the participant; however, even with this correction for audibility, some high-frequency speech components may not have been fully audible for the YOHL and OHL listeners (see Humes, 2007 and Smith et al., 2012), whereas the YN group did not require any correction because they had normal hearing. Thus, we conclude that these results overall did not provide compelling evidence to support our hypotheses that there would be significant associations between measures of working memory and speech understanding. One reason for the lack of correlations may be that the current study was underpowered with 24 participants per group. Future work should test a larger sample size. Another reason may be because the entire speech signal was not fully audible in the older groups, thereby preventing the contribution of working memory to speech understanding from being fully realized in those listeners.

In light of the absence of significant correlations between measures of working memory and speech understanding, the factor analysis was performed to determine if indeed the speech measures were distinct and if the memory measures overlapped with the measures of speech understanding. The factor analysis indicated that the LISN test of discourse comprehension was unique relative to the other measures of speech understanding, but that the remaining measures of speech understanding based on a simple repetition task were not distinguishable enough to load on separate factors. In essence, whether word-level or sentence-level materials were used, the measures that loaded on Factor 1 employed a simple immediate word repetition task. For example, for the QuickSIN and VAST tests, the task of the listener is to repeat the entire sentence, with the sentence being scored in terms of the number of keywords that are correctly repeated, whether or not the sentence that is repeated makes sense. For the multi-SNR R-SPIN, the whole sentence is presented, but the task of the listener is to repeat only the sentence-final word. Taken together, the findings that memory, repetition and comprehension measures were not correlated with each other and that they were not overlapping factors in the factor analysis, suggests that these factors are distinct and may depend as much if not more on task than on the linguistic nature of the test materials.

Another issue to consider is the ecological validity of using word recognition and comprehension measures as surrogates for everyday conversations. It could be that associations between memory and speech understanding measures would be significant if a more ecologically relevant measure of speech understanding, such as conversational fluency, were used rather than the relatively artificial and passive listening measures used in the current study. Additionally, in the present study, the measures used a mixture of materials spoken by different talkers and presented in quiet or in different types of babble. Future research examining the effects of linguistic complexity and task demands on the association between working memory and speech understanding should consider using a range of speech materials with the same talker in quiet and with consistent competing noise(s) to ensure that participants receive all levels of materials in all conditions with better control over the acoustic properties of the test materials. In addition, the effects of age and hearing loss may be better elucidated if groups of both younger and older adults with matched degrees of hearing thresholds (normal and with hearing loss) were used or if auditory performance was matched on the basis of other non-speech auditory measures of supra-threshold processing.

## Conclusion

In summary, the data showed that all participants had better performance with the auditory WARRM test than with the visual RS test, most likely because the WARRM sentences were linguistically simpler and demanded less processing compared to the sentences used in the RS test. In addition, variability in verbal working memory was observed when participants were tested with the auditory WARRM test than with the visual RS test, most likely because the WARRM test was more sensitivity to individual differences in auditory processing. Furthermore, the findings did not provide overwhelming evidence that working memory is associated with various measures of speech understanding in any of these listener groups, regardless of age or hearing status. Instead, the findings suggest that measures of memory, word recognition and discourse comprehension tap distinct abilities that may be related to everyday listening and that these abilities should be measured separately. Future studies should use more consistent materials and methodological approaches to elucidate a better understanding regarding the possible associations between inter-individual differences in working memory and speech understanding in more ecologically relevant conditions.

## Author Contributions

SS and MP-F both made contributions to the design of the work. SS oversaw data collection and analyzed the data. SS and MP-F both made contributions to interpretation of the work, drafting and revising the manuscript for important intellectual content, approving the final version to be published, and are accountable for all aspects of the work.

## Conflict of InterestStatement

The contents of this manuscript do not represent the views of the Department of Veterans Affairs or the United States government. The authors declare that the research was conducted in the absence of any commercial or financial relationships that could be construed as a potential conflict of interest.

## Footnotes

1 These groups of participants readily were available and were chosen to enable comparison to the common participant groups in prior studies. In particular, the two older groups with hearing loss are important because they were drawn from the clinical population of interest and provide a contrast in terms of age while matching on audiometric thresholds. The younger group with normal hearing provide an extreme contrast in terms of both age and hearing thresholds. We recognize that inclusion of a group of older listeners with normal hearing and a group of younger listeners with hearing loss would have offered a more ideal examination regarding the effects of age and hearing loss; however, older adults with normal hearing would not usually be seen in audiology clinics and the underlying mechanisms of hearing loss in younger adults are not the same as those of age-related hearing loss even though audiometric thresholds may similar.

2 Tinnitus information was available only for the older listeners via a chart review. The majority of older listeners (40/48) reported experiencing tinnitus in some way as an adult (e.g., history of tinnitus would be positive even if they reported tinnitus occurring rarely, or only for a few minutes, etc.) and only 17.5% (7/40) reported that their tinnitus was bothersome in some way (i.e., can interfere with sleep at times, etc.). There were no differences on the two primary memory measures as a function of tinnitus being: (1) positive vs negative history, (2) constant vs intermittent, or (3) bothersome vs not bothersome. Future studies in this area should consider tinnitus for inclusion/exclusion purposes.
